# Rat Adipose Tissue-Derived Stem Cells Transplantation Attenuates Cardiac Dysfunction Post Infarction and Biopolymers Enhance Cell Retention

**DOI:** 10.1371/journal.pone.0012077

**Published:** 2010-08-10

**Authors:** Maria E. Danoviz, Juliana S. Nakamuta, Fabio L. N. Marques, Leonardo dos Santos, Erica C. Alvarenga, Alexandra A. dos Santos, Ednei L. Antonio, Isolmar T. Schettert, Paulo J. Tucci, Jose E. Krieger

**Affiliations:** 1 Heart Institute, University of São Paulo Medical School, São Paulo, Brazil; 2 Radiopharmacy Laboratory, Nuclear Medicine Center, University of São Paulo Medical School, São Paulo, Brazil; 3 Biophysics Department, Federal University of São Paulo, São Paulo, Brazil; 4 Cardiology Division, Federal University of São Paulo, São Paulo, Brazil; University of Cincinnati, United States of America

## Abstract

**Background:**

Cardiac cell transplantation is compromised by low cell retention and poor graft viability. Here, the effects of co-injecting adipose tissue-derived stem cells (ASCs) with biopolymers on cell cardiac retention, ventricular morphometry and performance were evaluated in a rat model of myocardial infarction (MI).

**Methodology/Principal Findings:**

^99m^Tc-labeled ASCs (1×10^6^ cells) isolated from isogenic Lewis rats were injected 24 hours post-MI using fibrin a, collagen (ASC/C), or culture medium (ASC/M) as vehicle, and cell body distribution was assessed 24 hours later by γ-emission counting of harvested organs. ASC/F and ASC/C groups retained significantly more cells in the myocardium than ASC/M (13.8±2.0 and 26.8±2.4% vs. 4.8±0.7%, respectively). Then, morphometric and direct cardiac functional parameters were evaluated 4 weeks post-MI cell injection. Left ventricle (LV) perimeter and percentage of interstitial collagen in the spare myocardium were significantly attenuated in all ASC-treated groups compared to the non-treated (NT) and control groups (culture medium, fibrin, or collagen alone). Direct hemodynamic assessment under pharmacological stress showed that stroke volume (SV) and left ventricle end-diastolic pressure were preserved in ASC-treated groups regardless of the vehicle used to deliver ASCs. Stroke work (SW), a global index of cardiac function, improved in ASC/M while it normalized when biopolymers were co-injected with ASCs. A positive correlation was observed between cardiac ASCs retention and preservation of SV and improvement in SW post-MI under hemodynamic stress.

**Conclusions:**

We provided direct evidence that intramyocardial injection of ASCs mitigates the negative cardiac remodeling and preserves ventricular function post-MI in rats and these beneficial effects can be further enhanced by administrating co-injection of ASCs with biopolymers.

## Introduction

Transplantation of stem cells represents a promising approach for cardiac repair post-myocardial infarction (MI), but developing this therapeutic strategy for clinical use remains a challenge. A number of issues must be optimized, which include, but are not limited to, transplanting the appropriate type and numbers of cells, specific timing and route of cell delivery, developing improvements in cell retention and survival in the cardiac tissue, and identifying the detailed mechanisms of actions underlying the desired effects [1]. Replacement of cardiomyocytes will be required for restoring pumping capacity following large loss of tissue post-MI. However, this goal remains unmet and different cell types have been shown, especially in pre-clinical studies, to potentially prevent or delay further deterioration of cardiac function post-MI. Adipose tissue-derived stem cells (ASCs) have been considered for autologous adult stem cell transplantation procedures due to their abundancy, ease of sampling [2], [3], [4], and similarity in appearance to bone marrow cells (BMCs) [5], [6], [7], with a comparable potential to differentiate into diverse cell lineages [3], [8], [9], [10], [11], [12], [13], including endothelial cells [14], [15] and cardiomyocytes [16], [17]. Furthermore, recent studies have reported beneficial outcomes after administrating ASCs to treat myocardial infarction [18], [19], [20], [21], [22].

Given that a significant number of transplanted cells detach from the target tissue or die, during or soon after injection [23], [24], [25], [26], [27], diverse strategies are being intensely studied to improve cell adhesion and survival at the time of transplantation. *In situ* tissue engineering or the utilization of injectable biomaterials as vehicles to deliver cells directly into the target tissue may improve cell therapy by physically entrapping the cells at the injection site and by providing a favorable structural support for cell survival and proliferation [28]. Indeed, the combination of cells with biopolymers improves transplanted cell survival, induces angiogenesis, reduces infarct expansion, and preserves cardiac function after infarction [29], [30], [31], [32]. We demonstrated that injection of BMCs with fibrin 72 hours after infarction increases transplanted cardiac cell retention by 2.5-fold suggesting that injectable biopolymers diminish the peri-implant cell loss [32].

In the present study, we evaluated both the therapeutic potential of ASCs for cardiac repair, and the ability of two of the most used injectable biopolymers, fibrin and collagen, to improve ASC cardiac retention and functional outcomes after acute myocardial infarction.

## Materials and Methods

### Animal model of myocardial infarction

Experimental procedures followed the institutional guidelines for care and use of laboratory animals and were approved by the Institutional Review Board of the University of São Paulo Medical School, Brazil (#527/04). Ten-week-old female inbred Lewis rats were given rat chow diet and water *ad libitum* and housed under an alternating 12 hour/12 hour light/dark cycle. Experimental myocardial infarction was produced by ligation of the descending left coronary artery as previously described [33]. Briefly, a lateral thoracotomy was performed under anesthesia and the left coronary artery was looped by a single nylon suture (5.0) at approximately 1 mm from its origin and gently tied. This procedure produced a clearly demarcated area (cyanotic and bulging) of acute ischemia corresponding to the distribution of the left coronary artery distal to the occlusion. The chest was closed and rats were individually caged during a 24 hour recovery period.

### Cell Isolation and *ex vivo* Expansion

Inguinal subcutaneous adipose tissue was collected under sterile conditions from ten-week-old male Lewis rats and rinsed with phosphate-buffered saline (PBS). ASCs were isolated, characterized and maintained in culture as previously described [34], [35]. Under this experimental condition, the cells are homogeneous and express the surface markers CD90 and CD 29 and display adipogenic and osteogenic differentiation potential. In brief, harvested tissue was enzymatically dissociated and centrifuged. Pelleted cells were recovered and plated onto 10 cm culture plates. At 24 hour intervals, cultures were washed with PBS to remove contaminating erythrocytes and other unattached cells, and refed with fresh medium. This process was repeated for three days. Plating and expansion medium consisted of Dulbecco's modified Eagle's medium (DMEM) low glucose with 10% Fetal Bovine Serum (FBS) and 1% (v/v) penicillin–streptomycin (1000 UI/mL–1000 µg/mL; GIBCO BRL, Gaithersburg). Cells were maintained at 37°C with 5% CO_2_ in tissue culture dishes or flasks and fed twice per week. Cells reached 80% confluence within 5–7 days after the initial plating (passage 0). At 80% confluence, adherent cells were detached with 0.05% trypsin-EDTA and re-plated at 1.0×10^4^ cells/cm^2^. Cultures were passaged every 3–5 days and used for experimental procedures at passage 3 and 4.

### Biopolymers isolation and preparation

The fibrin polymer was prepared by combining fibrinogen and thrombin at the time of injection. Fibrinogen was obtained from plasma separated from 50 ml rat whole blood and 3.8% sodium citrate (5 ml) was added. Fibrinogen was isolated using a cryoprecipitation technique [36] and diluted to a final concentration of 300 mg/dl. Human thrombin (Baxter Healthcare Inc.; Norfolk, UK) was used to catalyze fibrin polymerization. ASCs were resuspended in 80 µl of the fibrinogen solution and 20 µl of thrombin (250 UI/ml) was added to the syringe containing the cell suspension a few seconds before myocardium injection. This combination allowed for a suitable window of time (20 seconds) to perform the myocardial injection prior to polymerization.

Collagen was isolated from rat tail tendons using the method described by Habermehl *et al.* [37] with slight modifications. Briefly, tail tendons were extracted under sterile conditions, rinsed in deionized water and dissolved in 1% acetic acid (v/v). After 72 hours at 4°C, the solution was centrifuged at 30,000*×g* for 2 hours and the supernatant collected and stored at the same temperature. Before *in vitro* and *in vivo* assays, the acid soluble collagen solution was neutralized by mixing with 0.1 N sterile solution of NaOH in water, and the final concentration was adjusted to 3 mg/ml. The physiological pH at 37°C resulted in jellification of collagen.

### Cellular viability assay

ASC proliferation/viability was evaluated by the 3-(4,5-dimethylthiazol-2-yl)-2,5-diphenyltetrazolium bromide (MTT) assay for up to 4 days, based on the reduction of tetrazolium salt to formazan crystals by living cells, as previously described [38]. Approximately 170 µl of MTT solution (5 mg/ml) was added to each well. After two hours, cell morphology was analyzed by inverted optical microscopy, and formazan salts were dissolved with 10% SDS/0.01 N HCl. The mixture was incubated for 18 hours, and optical density (O.D.) measurements were performed at 540 nm (ELX 800, Biotek).

### ASC radiolabeling

Ceretec® lyophilized kit (GE Healthcare; Cardiff, UK) was reconstituted in 2 ml of 0.9% NaCl solution containing 1.48 GBq (40 mCi) of sodium pertecnetate (IPEN-CNEN, Brazil). Radiochemical purity of the labeled product [technetium 99m–hexamethylpropylene amine oxime (^99m^Tc-HMPAO)] was determined by ethyl acetate/saline extraction procedure. ASCs were labeled with ^99m^Tc-HMPAO, as previously described for unfractionated BMCs [32]. Briefly, the suspension of ASCs was centrifuged and the pellet was resuspended in 1 ml of ^99m^Tc-HMPAO solution and incubated for 15 minutes at 37°C. Plasma was added to interrupt cell tagging and the suspension was centrifuged at 466*×g* for 10 minutes. The supernatant was discarded and the pellet resuspended in PBS. The centrifugation and suspension procedure was repeated. Labeling efficiency, calculated as the ratio of the activity in ^99m^Tc-labeled ASCs to the total radioactivity was 15%.

To evaluate ASC labeling stability and nonspecific retention of free tracer into the biopolymer mesh, 1×10^5^ radiolabeled cells were plated, incubated for 24 hours and separated from the culture medium by centrifugation. Radioactivity was measured in the pellet and supernatant, and expressed as the ratio of the radioactivity in each fraction to the total radioactivity (radioactivity in cells plus in supernatant). Similar to previously published for BMCs [32], only 30.1±0.3% of the radioactivity initially incorporated by the ASCs remained within the cells 24 hours after labeling. Since no significant deterioration of cell viability was detected by the trypan blue dye exclusion test, in the present study, radioactivity values were also corrected according to the rate of ^99m^Tc leakage from ASCs *in vitro* (fixed at 30.0%) assuming an equivalent rate of leakage *in vivo*.

### Cell transplantation

Twenty-four hours after the MI, animals were subjected to a second surgical procedure for a single transepicardial injection of 1×10^6^ ASCs suspended in 100 µl of one of the following vehicles: serum-free DMEM culture medium (group ASC/M), fibrin (group ASC/F) or collagen (group ASC/C). Controls receiving same volumes of cell-free media (group M), fibrin (group F) or collagen (group C) were also injected. All injections were performed within the infarct border zone of the anterior left ventricular free wall using a 30-gauge needle. False-infarcted animals (Sham group) were used as normal animals for cardiac morphometry and function. Please note that there was no attempt to select animals with comparable MI areas prior cell transplantation (24 hours after coronary ligation), so only animals with comparable MIs, four weeks later, were analyzed for the different phenotypes. This precludes the assessment of the treatments on the MI size, but allows a careful estimate of the interventions on the different phenotypes in animals with comparable MI areas. The 24 hour time-point was selected to avoid immediate post-MI acute inflammatory phase that may contribute to increase cell death and decrease cell engrafting and due to the fact that cardiac cell retention appears to be similar within the 24–72 hour post-MI[32].

### Nuclear radiometry of harvested organs

Animals that received radiolabeled ASCs were euthanized 24 hours after cell transplantation and heart, lungs, liver, spleen, kidneys, femur and a sample of blood (approximately 3 ml) were harvested and weighed. Radioactivity levels of the whole isolated organs were determined in a gamma counter (Automatic Gamma Counter 1480 – Perkin Elmer; Shelton CT, USA). Radioactivity values of blood and bones were estimated from the amount of radioactivity in samples of these tissues, considering them as 7% and 10%, respectively, of the mass of the entire animal [39]. The results were expressed as a percentage of total injected radioactivity after correction for radioactive decay.

### Morphometric analyses and capillary density determination

At the end of experimental procedures, hearts were quickly removed and fixed in 10% formalin for 24 hours, embedded in paraffin, and histologically sectioned (5 µm). Samples were mounted onto slides and stained with Picrossirius Red for measurement of the following morphometric parameters: (i) average scar size and (ii) thickness, (iii) left ventricular wall perimeter, (iv) average thickness of the interventricular septum and (v) the percentage of its area occupied by interstitial collagen. Capillary densities were evaluated at the infarct border zones of samples stained with Periodic Acid of Schiff (PAS), and expressed as the mean number of capillaries in the area of the fields (mm^2^) at 400× magnification. All procedures were performed in samples obtained from middle and apical transversal segments of the heart and results were expressed as the mean of the values from both segments. Images were obtained by an image acquisition software system (NIS - Elements AR 2.30) and measurements were determined by ImageTool® software (UTHSCSA). Infarct size was estimated as the percentage of left ventricular perimeter containing scar tissue.

All measurements were performed by two independent investigators blinded to the experimental treatments.

### Global cardiac function by direct hemodynamic evaluation

Thirty days after MI, invasive hemodynamic studies were performed on a heated rodent operating table (37°C) under adjusted anesthesia (urethane 1.2 g/kg), and oxygen-enriched mechanic ventilation. Left femoral vein was accessed to supplement anesthesia, drugs or saline. Bilateral vagotomy was performed to prevent changes in heart rate values induced by the baroreflex. A Millar micro manometer (MikroTip™ 2F, Millar Instruments Inc., Houston, TX, USA) was inserted from the right carotid artery to the LV cavity to access intraventricular pressure. A blood flow ultrasound probe (Transonic Systems Inc. NY, USA) was positioned on the ascending aorta to access stroke volume (SV) (excluding coronary flow) through right thoracotomy. Data were acquired by the software Acknowledge (Biopac Systems, CA, USA) to measure systolic (LVSP) and end-diastolic left ventricle pressures (LVEDP), heart rate (HR), cardiac output (CO) and SV. Stroke work (SW) was estimated offline as a product of SV×developed pressure (LVSP – LVEDP)×constant 0.0136.

Hemodynamic parameters were determined under basal conditions and during a sudden pressure-overload with a vasoconstrictive phenylephrine (PHE) bolus injection (25–75 µg/kg body weight) into the left femoral vein [40]. PHE doses were adjusted for individual animals to produce comparable elevations in blood pressure (60% to 80% greater than baseline).

### Statistical analysis

Results are expressed as mean ± standard error of the mean (SEM). One- or two-way analysis of variance (ANOVA) with Turkey's *post-hoc* test, or unpaired Student's *t* test was utilized to compare groups, when appropriate. All statistical analyses were performed using GraphPad Prism 4.0 (GraphPad Softwares Inc., CA, USA). *P* values≤0.05 were considered significant.

## Results

### Viability of ASCs cultured in biopolymers

**Figure 1:**
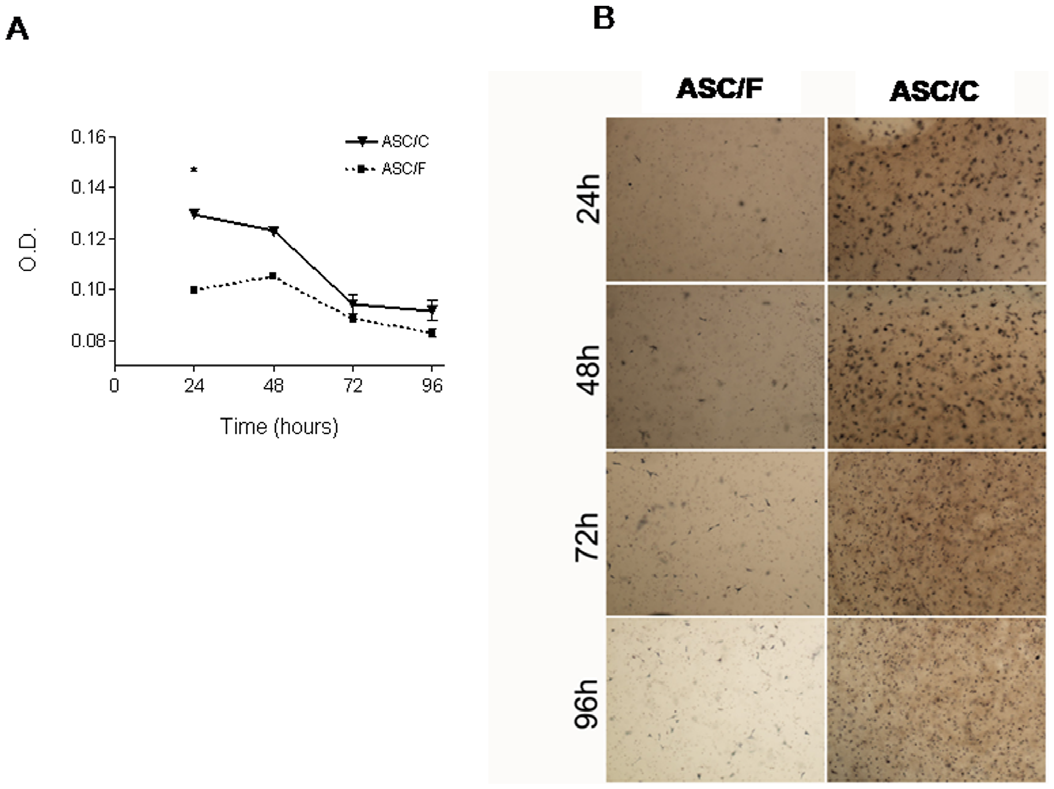
Cell viability on 3D biopolymer scaffolds. (A) Variation of optical density (O.D.) with time in culture. The O.D. at 540 nm indicates cell viability. *, *P*<0.05 *vs.* ASC/F. (B) Microphotographs of representative cultures after MTT stain. Magnification 40×. ASC/F, cells cultured in fibrin (n = 6); ASC/C, cells cultured in collagen (n = 6).

To assess differential effects of biopolymers on cell survival, we investigated the viability of ASCs seeded *in vitro* in three-dimensional (3D) scaffolds of fibrin and collagen for up to 4 days using the MTT assay. The O.D. used as an index of cell density at 24 hours post seeding was significantly higher in ASCs seeded in collagen compared to fibrin (ASC/C 0.1295±0.8×10^−3^ *vs.* ASC/F 0.0995±1.57×10^−3^; *P*<0.05) denoting higher viability (figure 1A). Optical density values gradually decreased in ASCs cultured in both biopolymers as expected in cells seeded in scaffolds, as a consequence of changes in mechanical properties which in turn affects cell growth [41]. Figure 1B shows representative microphotographs of MTT stained plates across time.

### ASC cardiac retention and biodistribution

**Figure 2:**
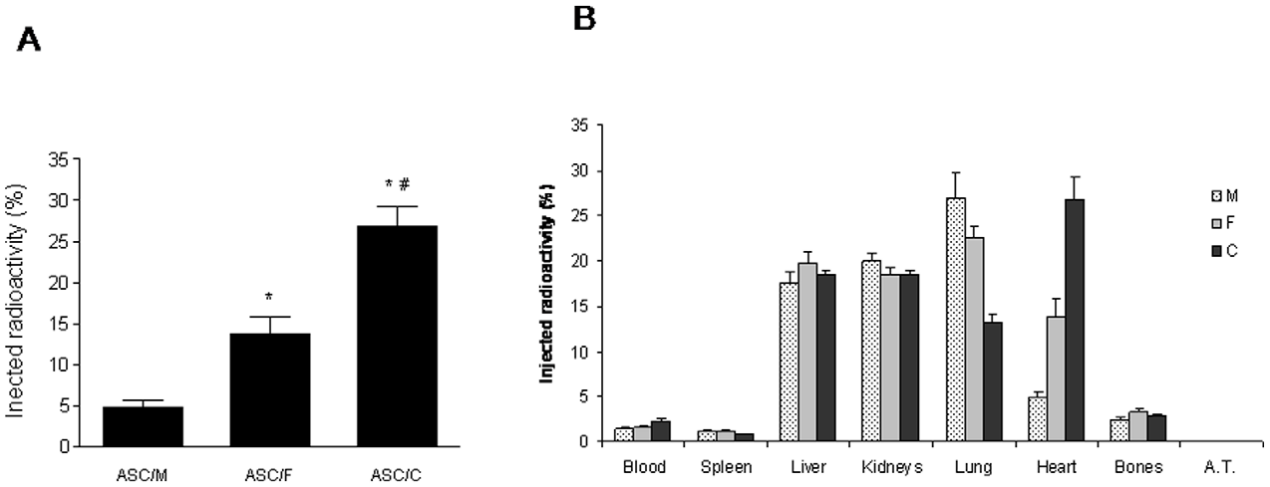
Effects of injectable biopolymers on ASC cardiac retention. (A) Cardiac retention of ASCs 24 hours after intramyocardial injection using either culture medium (ASC/M, n = 12), fibrin (ASC/F, n = 13) or collagen (ASC/C, n = 16). (B) Distribution of radioactivity in different organs according to the vehicle used to administer the radiolabeled ASCs into the myocardium. Data are expressed as percentage means (±SEM). *A.T.*, adipose tissue. *, *P*<0.05 *vs.* ASC/M; #, *P*<0.05 *vs.* ASC/F.

To determine the biopolymer with the best profile in terms of cardiac cell retention, radiolabeled ASCs were injected into the infarct border zone 24 hours post-infarction using either fibrin (group ASC/F), collagen (group ASC/C) or culture medium (group ASC/M) as vehicle, and radioactivity was assessed in several tissues 24 hours after injection. As expected, both biopolymers significantly increased ASC cardiac cell retention compared to culture medium (figure 2A). The ASC/C group showed the highest radioactivity retention (26.8±2.4%), which nearly doubled the retention observed for the ASC/F group (13.7±1.9%, *P*<0.05 *vs.* ASC/C) and five-folded the value observed for ASC/M (4.84±0.7% *vs.* ASC/C, *P*<0.05). To examine the possibility that the tracer that leaked from the cells was nonspecifically retained in the biopolymer mesh, radiolabeled ASCs were incubated either into biopolymer scaffolds (ASC/C and ASC/F) or as adherent cultures (ASC/M) for 24 hours and radioactivity was measured in both the cell and the supernatant fractions. The amount of radioactivity in the cell fraction was highly similar for all groups (30.6±3.4; 29.5±4.5 and 30.2±2.2% for cells cultured in M, F and C respectively; *P*>0.05), indicating that it is unlikely tracer leaked from the cells, remained bound to the biopolymers, and interfered with the results obtained.

Interestingly, the distribution of radioactivity among several tissues 24 hours after injection showed that most labeled cells did not remain in the heart. An inverse relationship was observed between cardiac ASC retention and lung retention (figure 2B). These results suggest that ASCs which are washed out from the myocardium through the lymphatic vessels and veins from the left ventricle (LV) may ultimately be delivered into lung tissue. High radioactivity levels were also detected in liver and kidney tissues, and markedly lower values were noted in spleen, bones and adipose tissue.

### Therapy effects on heart morphometry

**Figure 3:**
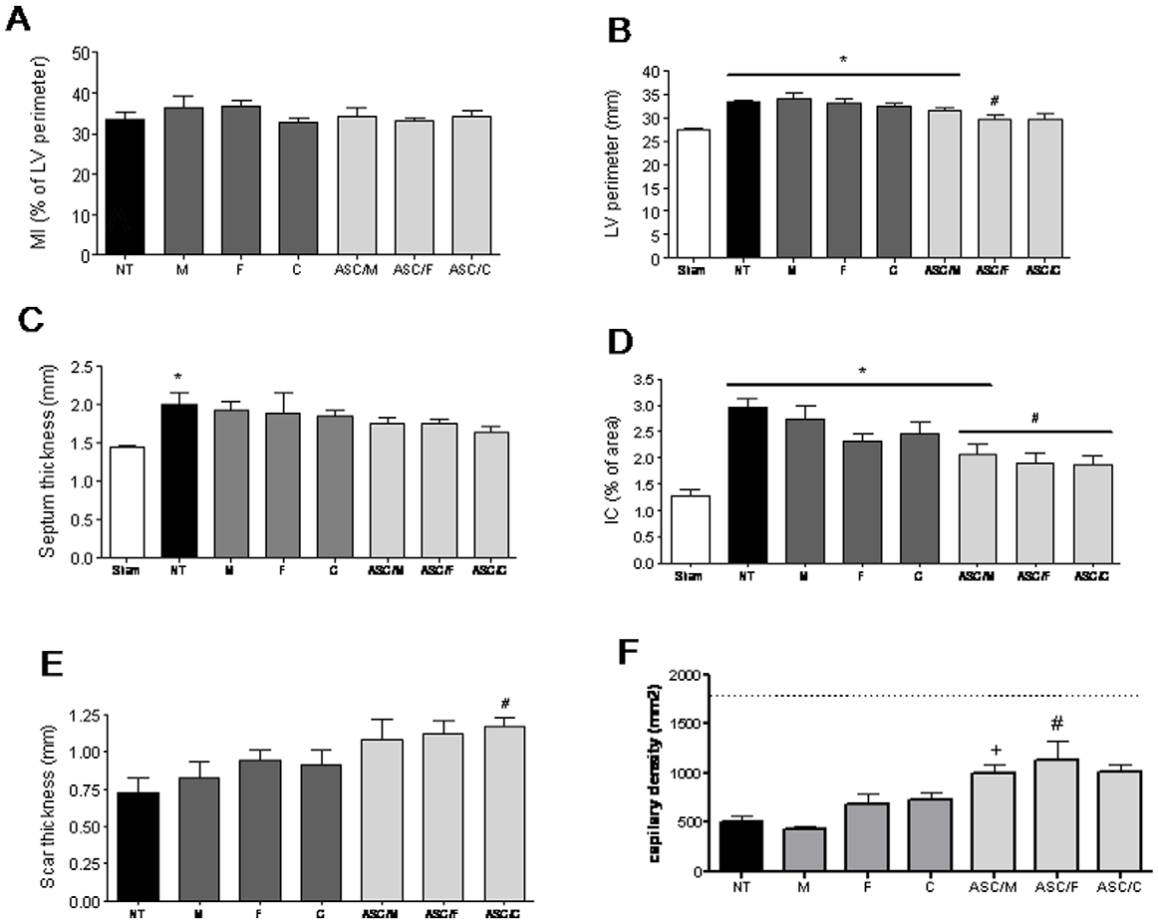
Cardiac morphometry 4 weeks after treatment. (A) Myocardial infarct size estimated as the percentage of left ventricle perimeter occupied by scar. (B) Left ventricle perimeter expressed in millimeters. (C) Interventricular septum thickness in millimeters. (D) Fibrosis in spare myocardium, expressed as percentage of septum area occupied by interstitial collagen. (E) Scar thickness in millimeters. (F) Capillary density, as the mean of number of capillaries per area (mm^2^; magnification 400×). Note that Sham capillary density averages 1793 capillaries/field as shown in the traced line. MI, myocardial infarct size; LV, left ventricle; IC, interstitial collagen; Sham, (n = 7); NT, non-treated (n = 8); M, culture medium (n = 5); F, fibrin (n = 6); C, collagen (n = 6); ASC/M, ASCs in culture medium (n = 6); ASC/F, ASCs in fibrin (n = 7); ASC/C, ASCs in collagen (n = 6). *, *P*<0.05 *vs.* Sham; *#*, *P*<0.05 *vs.* NT; +, *P*<0.05 *vs.* respective vehicle control.

To assess whether the enhanced cardiac retention of ASCs influenced the deterioration normally observed in the left ventricle, morphometric parameters indicative of ventricular remodeling were analyzed in the experimental groups 4 weeks after MI (shown in table 1). As depicted in figure 3A, no differences in infarct size among groups were observed (33.4±1.8; 36.3±2.9; 36.6±1.5; 32.7±1.2; 34.3±1.9; 33.0±0.7 and 34.0±1.4% for NT, M, F, C, ASC/M, ASC/F and ASC/C respectively; *P*>0.05). This finding can be explained by the pre-established selection criteria in which only animals with infarcts ranging from 30 to 40% of the LV perimeter at the end of the protocol were included in the analyses. Indeed, ligation of the left anterior descending coronary artery results in large variation in MI size. For this reason, animals with comparable MI dimensions may be selected by echocardiography prior to treatment randomization or, alternatively, animal randomization may initially occur followed by selection of animals with comparable MI dimensions at the end of the protocol. Although results cannot be used to evaluate MI size in the case of the latter example, results can shed light on the influence of the treatments in cardiac structure and function at a period of 4 weeks post-MI.

**Table 1:** Experimental groups.

Group	MI	Content	n
Sham	−	-	7
NT	+	-	8
M	+	Medium	5
F	+	Fibrin	6
C	+	Collagen	6
ASC/M	+	1×10^6^ ASCs+medium	6
ASC/F	+	1×10^6^ ASCs+fibrin	7
ASC/C	+	1×10^6^ ASCs+collagen	6

*MI*, myocardial infarction; *n*, number of animals in each group; *Sham*; false infarcted; *NT*, non-treated; *M*, culture medium; *F*, fibrin; *C*, collagen; *ASCs*, adipose tissue-derived stem cells; *ASC/M*, ASCs in culture medium; *ASC/F*, ASCs in fibrin; *ASC/C*, ASCs in collagen.

In groups NT, M, F, C and ASC/M, the perimeter of the LV was significantly increased compared to the Sham group (33.5±0.2; 34.0±1.1; 33.1±0.8; 32.4±0.5 and 31.5±0.6 mm for NT, M, F, C and ASC/M respectively *vs.* 27.4±0.2 mm for Sham; *P*<0.05) as a consequence of the negative remodeling effect of the infarct (figure 3B). Conversely, in groups ASC/F and ASC/C, LV perimeter was preserved showing an overall smaller ventricle size (29.5±3.3 mm and 29.7±1.0 mm, respectively) and the LV perimeter values for group ASC/F were lower than that observed for the NT group (*P*<0.05).

Increase in septum thickness, an indicator of cardiac hypertrophy, remained unchanged in the ASC-treated groups. A tendency for increased septum thickness was noted in the control groups following the MI, but reached significance in the NT group compared to Sham group (2.0±0.1 *vs.* 1.4±0.0 mm for NT *vs.* Sham; *P*<0.05) (figure 3C).

Fibrosis in the spare myocardium, measured as the percentage of the septum area occupied by interstitial collagen, was significantly higher in NT, M, F, C and ASC/M groups when compared to Sham (2.9±0.1; 2.7±0.2; 2.3±0.1; 2.4±0.2; 2.0±0.1% of septum area for NT, M, F, C and ASC/M respectively *vs.* 1.2±0.1% for Sham; *P*<0.05) (figure 3D). Moreover, the groups that received ASCs, associated or not with biopolymers, showed lower percentages of interstitial collagen than the NT group (2.0±0.1; 1.9±0.1; and 1.8±0.1% for ASC/M, ASC/F and ASC/C respectively *vs.* 2.9±0.1% for NT; *P*<0.05). Similarly, the scars tended to be thicker in ASC-treated (figure 3E), but reached significance only in the ASC/C compared to NT group (1.2±0.0 mm for ASC/C *vs.* 0.7±0.0 mm for NT; *P*<0.05).

The capillary density in the infarct border zone tended to be higher in the cell-treated groups, but reached significance only in the ASC/F compared to group NT (1139±194.3 *vs.* 500±58.9 capillaries/mm^2^ for ASC/F and NT respectively; *P*<0.05) (figure 3F and Figure S1). It is important to notice that four weeks after cell transplantation, there was no evidence of lasting polymers in the areas of cardiac tissue where labeled injected cells were present (Figure S2 and Methods S1).

### Therapy effects on cardiac function

Cardiac performance was measured by direct hemodynamic assessment carried out under both basal conditions and conditions associated with a pharmacologic challenge upon injection of a potent vasoconstrictor, phenylephrine. Under basal conditions, indices of ventricular function in infarcted animals were worse than Sham animals (table 2). While LVSP diminished in group M, LVEDP increased in groups NT and C.

**Table 2:** Hemodynamic variables under basal conditions.

	Sham	NT	M	F	C	ASC/M	ASC/F	ASC/C
LVSP (mm Hg)	119.6±4.6	116.6±4.1	101.4±1.9 *	109.7±3.4	115.5±3.2	112.8±2.5	110.9±2.1	114.5±3.7
LVEDP (mm Hg)	4.7±1.2	13.0±1.5*	11.6±1.1	10.3±0.8	11.8±3.2 *	8.5±0.4	9.0±1.0	7.6±1.0
HR (bpm)	356±31	328±12	365±16	396±12	374±20	378±11	364±11	358±16
+dP/dt (mm Hg/s)	9991±464	7124±285*	6019±366*	7230±438 *	8072±415* †	8563±474†	7935±267*	8906±557†
−dP/dt (mm Hg/s)	−6741±430	−5133±178*	−4449±148*	−5476±247	−5691±354	−5872±159	−5451±218	−6240±469
CI (ml/kg/min)	190±14	201±6.9	183±18	154±4.7	201±19	160±18	191±8.7	170±19
SVI (ml/kg/beat)	0.490±0.052	0.596±0.051	0.498±0.057	0.392±0.014	0.598±0.067	0.424±0.045	0.410±0.049	0.485±0.066
SW (g·m/beat)	0.664±0.116	0.928±0.074	0.614±0.072	0.531±0.028	0.800±0.081	0.601±0.063	0.659±0.089	0.698±0.091

*LVSP*, left ventricular systolic; *LVEDP*, left ventricular end-diastolic pressures; *HR*, heart rate; *CI*, cardiac ejection index; *SV*, stroke volume index; *SW*, stroke work index. *Sham*, false operated; *NT*, non-treated; *M*, culture medium; *F*, fibrin; *C*, collagen; *ASC/M*, ASCs in culture medium; *ASC/F*, ASCs in fibrin; *ASC/C*, ASCs in collagen.

*P<0.05 vs. Sham.

†*P*<0.05 *vs.* M. One-way ANOVA followed by Tukey's *post-hoc* test.

**Figure 4:**
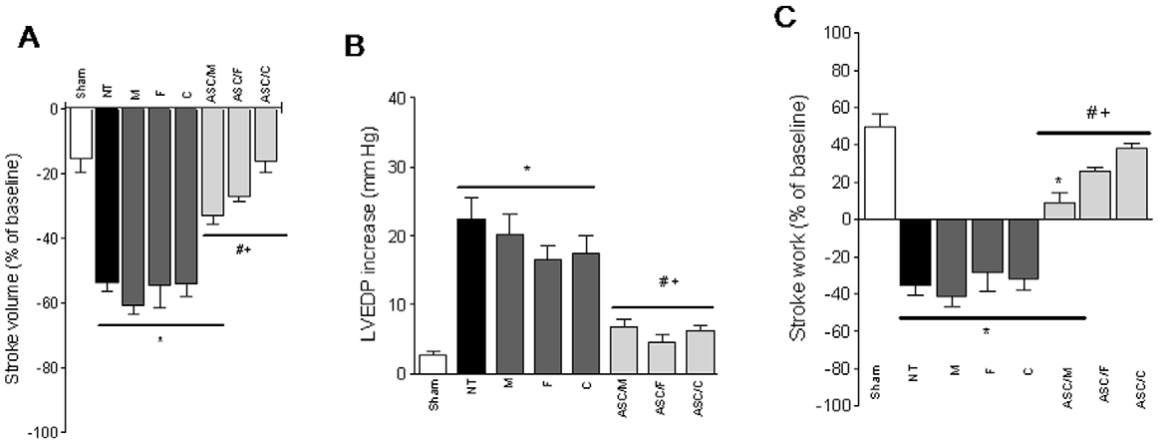
Cardiac response to afterload stress. Bars represent repercussions induced by phenylephrine injection, expressed as % of change from baseline (except for LVEDP, expressed as mm Hg elevation over baseline), on the stroke volume, LV end-diastolic pressure (LVEDP) and stroke work of Sham (n = 7), NT (n = 8), M (n = 5), F (n = 6), C (n = 6), ASC/M (n = 5), ASC/F (n = 7), and ASC/C (n = 6) groups. Positive or negative values resulted from increase or decrease on the evaluated parameter, respectively. *, *P*<0.05 *vs.* Sham; *#*, *P*<0.05 *vs.* NT; *+*, *P*<0.05 *vs.* respective vehicle control.

Upon phenylephrine injection, the cardiovascular phenotypic changes became much more evident, underscoring the improvement in the ASCs treated groups (figure 4). The degree of change on SV (amount of blood pumped from the ventricle in each cycle) in response to pressure overload was better preserved in the ASC groups compared to the NT group, and significantly different than their respective vehicle controls (−33.0±2.9; −27.0±1.7 and −16.2±3.6 *vs.* −53.7±2.5% for ASC/M, ASC/F and ASC/C *vs.* NT; and *vs.* −60.6±3.0; −54.5±6.9 and −54.1±3.8% for M, F and C; *P*<0.05) (figure 4A). The LVEDP values increased significantly post-MI in NT, M, F and C groups, whereas all ASC-treated groups exhibited pressure responses equivalent to the Sham animals which were significantly lower than both NT and their vehicle control groups (22.3±3.0; 20.2±2.9; 16.5±2.1; 17.3±2.6; 6.6±1.2; 4.5±0.9; 6.2±0.8 and 2.7±0.4 mmHg increase compared to baseline values for NT, M, F, C, ASC/M, ASC/F ASC/C and Sham respectively; *P*<0.05 for differences *vs.* Sham and *vs.* NT, *P*<0.05 for ASC/M and ASC/F *vs.* respective vehicle controls, and *P*<0.05 for ASC/C *vs.* C) (figure 4B). Finally, stroke work, a global index of cardiac function that depends on both pressure generation and ejection capability during each beat, displayed comparable profile in ASC-treated and Sham groups, with the exception of the ASC/M group which was injected in absence of any biopolymer and showed a significant difference from Sham (9.1±5.5; 25.7±2.1 and 38.0±2.8% change from baseline, for ASC/M, ASC/F and ASC/C, respectively *vs.* X±Y% for Sham; *P*<0.05 for ASC/M vs. Sham) (figure 4C). In contrast, all control groups showed negative changes in work generation in response to pharmacologic stress post-MI (−31.5±4.7%, −45.0±5.5, −27.5±10, and −23.0±6.2% for NT, M, F, and C, respectively. *P*<0.05 for all controls vs. Sham and ASC treated groups) (figure 4C).

### Correlation between ASC cardiac retention and cardiac performance

**Figure 5:**
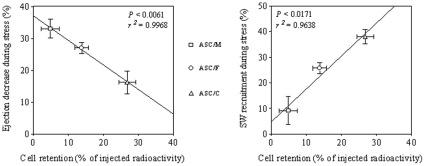
Dose-dependent effects of ASCs in heart function. Relationship between cell retention in the host myocardium (radioactivity of labeled ASC assessed 24 hours after injection) and cardiac performance (response to sudden afterload stress in terms of heart ejection (A) and stroke work (B) assessed 4 weeks after injection) of the infarcted hearts injected with ASC in culture medium (ASC/M), fibrin (ASC/F) or collagen (ASC/C). For radioactivity assay, samples were ASC/M = 12, ASC/F = 13, and ASC/C = 16; and for hemodynamic study, samples were ASC/M = 6, ASC/F = 7, and ASC/C = 6.

To obtain further evidence that ASC cardiac retention (determined as the percentage of total injected radioactivity remaining in the heart 24 hours post-injection) and improvement of cardiac performance post-MI (determined as the response to sudden afterload stress assessed 4 weeks after injection) may be linked, we performed a linear regression analysis. A significant correlation between ASC cardiac retention and both maintenance of stroke volume (r^2^ = 0.9968; *P*<0.05) (figure 5A) and increment of stroke work generation during the phenylephrine-induced afterload stress (r^2^ = 0.9638; *P*<0.05) (figure 5B) was demonstrated.

## Discussion

In the present study, we provided evidence that intramyocardial injection of ASCs 24 hours post-MI, delivered with culture medium, fibrin or collagen, significantly preserves left ventricular function, as assessed by direct hemodynamic evaluation. A correlation between cell retention capacity and preservation of stroke volume and capacity to generate cardiac work in response to pharmacologic stress was shown suggesting that these beneficial effects are proportional to cell number retention (figure 5). Unlike previously published studies [29], [42], under the present experimental conditions, the biopolymers *per se* did not show any effect on cardiac morphometry or function. Instead, the main effect of the biopolymers appeared to propagate an increase in local entrapment and/or survival of the transplanted cells.

Our data confirmed recent reports showing that transplantation of ASCs can favorably affect cardiac function after both acute and chronic infarction [18], [19], [20], [21]. Here, however, we further extended previous observations in three significant ways. First, we tested the therapeutic potential of ASC in combination with two important biopolymers for cell transplantation and tissue engineering, fibrin and collagen. These injectable and biodegradable scaffolds were shown here, as well as in previous studies [29], [30], [31], [32], [42], to increase engraftment of transplanted cells and to preserve cardiac function after infarction. Second, we assessed the cardiac retention of ASCs 24 hours after injection with different delivery vehicles. This approach allowed for the evaluation of the cell retention capacity of the tested biomaterials, to correlate degree of ASC retention with degree of heart function preservation, and to determine the main organs that cells spread. Third, we evaluated ventricular function by performing stringent invasive hemodynamic tests that allowed for direct measurements of ventricular pressure and aortic blood flow.

Interestingly, the favorable outcomes on cardiac function that we observed in this study were not manifested primarily by changes in resting or basal hemodynamic parameters but rather, in the altered cardiac response to stimuli such as sudden afterload stress induced by phenylephrine. A possible explanation for this is the rigorous inclusion criterion of animals with MI size of a limited range. While different infarct sizes may or may not affect cardiac function receiving cell therapy [43], in the present study, the selection criterion precluded assessment of the different treatments on infarct expansion. This approach revealed an important influence of ASC treatment on cardiac performance in MIs of comparable sizes four weeks after the insult. A direct relationship between the percentage of cardiac cells retained and the degree of cardiac function preservation post-MI was demonstrated, suggesting a dose effect for ASCs. Similar beneficial effects in cardiac performance post-MI were observed when we injected bone marrow cells (BMCs); however, no dose effect was detected for BMCs under the same conditions tested here [32], [44].

Although acute cardiac retention of ASC is markedly low, our data demonstrate that it can be significantly improved when cells are delivered in association with fibrin and collagen. For example, the intramyocardial co-injection of radiolabeled ASCs and collagen increased the amount of radioactivity five-fold in the infarcted heart at 24 hours following injection. In addition, we showed that the increased radioactivity retention observed when ASCs were delivered with fibrin and collagen were unlikely due to nonspecific trapping of free radiotracer in the biopolymers mesh, further confirming our previous results [32]. As expected, the nuclear radiometry of the harvested organs revealed a broad biodistribution of radiolabeled ASCs, with high radioactivity values detected in liver, kidney and lung tissues, and markedly lower values in spleen and bone tissues. In addition, for each delivery vehicle employed, we observed an inverse relationship between radioactivity accumulation in the heart and lungs. The noticeable ASC entrapment into the pulmonary circulation suggests that, excluding cell death, the major mechanism of cell loss is due to the washout from the myocardium through lymphatic vessels and veins of the left ventricular wall and may be associated with the time delay for the liquid carriers to fully polymerize. It was noteworthy that the biopolymers tested in this study do not compromise the viability of ASCs *in vitro* and do not elicit cardiac arrhythmias *in vivo* (data not shown).

The mechanisms by which ASCs may exert their beneficial effects deserve to be further explored; however, this is beyond the scope of this study. Based upon the observed improvements in terms of fibrosis attenuation and the overall preservation of left ventricular shape and size, which in turn improve function [45], [46], [47], we speculate that one of the underlying mechanisms may be the activation and/or potentiation of endogenous myocardial healing programs by paracrine means. This idea is supported by data reported in previous studies [18], [48], [49]. In addition, our data also revealed both an increase in capillary density among the ASC-treated groups and a tendency for an increase in scar wall thickness. While the former can have beneficial effects on cell preservation and cardiac function, the latter may provide mechanical support for the infarcted area improving overall cardiac function.

Taken together, the main finding of the present study is that intramyocardial injection of ASCs mitigates the negative cardiac remodeling and preserves ventricular function post myocardial infarction. In addition, we demonstrated that the beneficial effects on cardiac function can be further enhanced by administrating cells along with injectable biopolymers. Consequently, the results reported here may have important implications for the design of future cell therapy strategies for cardiac repair.

## Supporting Information

Figure S1 PAS staining for capillary quantification. Arrowhead indicates a single capillary in the heart sections from different groups. NT, non-treated; M, culture medium; F, fibrin; C, collagen; ASC/M, ASCs in culture medium; ASC/F, ASCs in fibrin; ASC/C, ASCs in collagen. Magnification 400×.
(2.04 MB TIF)

Figure S2 Immunodetection of GFP+ASCs four weeks after transplantation. Upper panel (A, B and C): immunohistochemistry. Bottom panel (D, E and F): immunofluorescence at infarction border zone. Arrowheads show the GFP+ASCs. Magnification 400×.
(2.04 MB TIF)

Methods S1 (0.02 MB DOC)
