# Supplementary figures and images for: Rat Adipose Tissue-Derived Stem Cells Transplantation Attenuates Cardiac Dysfunction Post Infarction and Biopolymers Enhance Cell Retention
Source: PLoS One. 2010 Aug 10;5(8):e12077. doi: 10.1371/journal.pone.0012077 (PMC2919414; doi:10.1371/journal.pone.0012077)

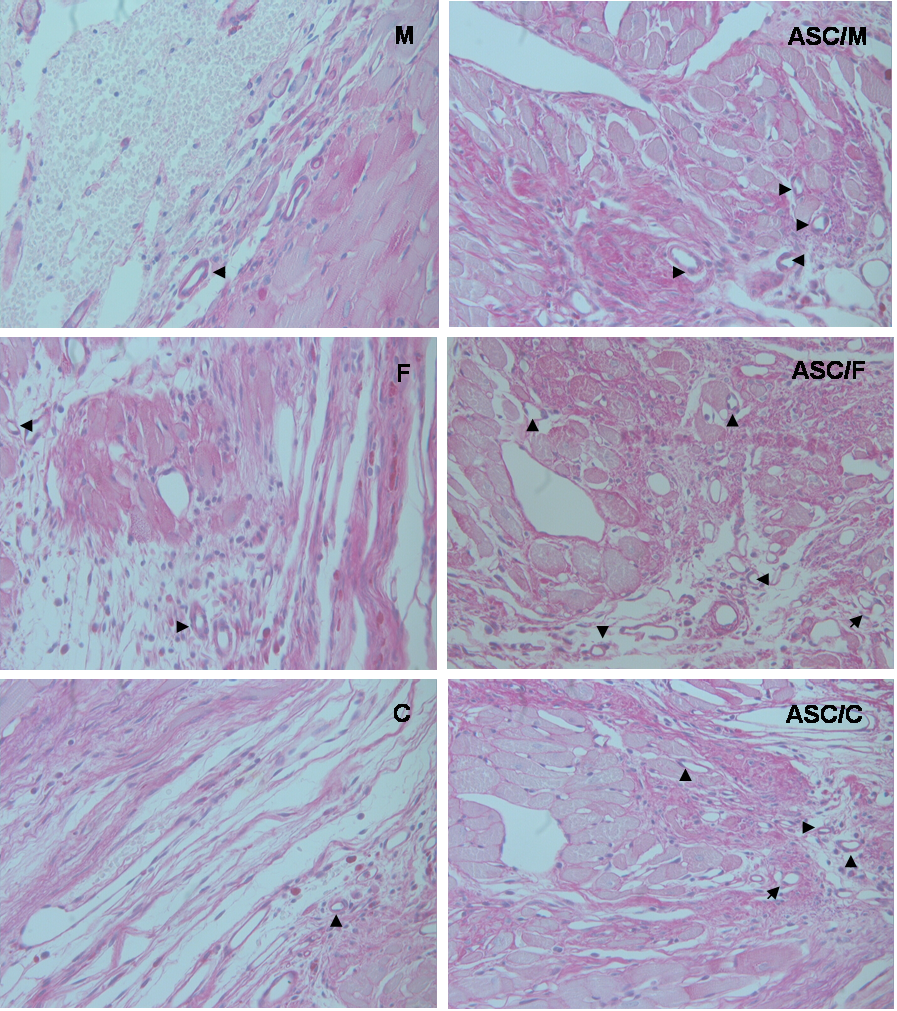

Supplement: Figure S1 — PAS staining for capillary quantification. Arrowhead indicates a single capillary in the heart sections from different groups. NT, non-treated; M, culture medium; F, fibrin; C, collagen; ASC/M, ASCs in culture medium; ASC/F, ASCs in fibrin; ASC/C, ASCs in collagen. Magnification 400×. (2.04 MB TIF) [file pone.0012077.s001.tif]

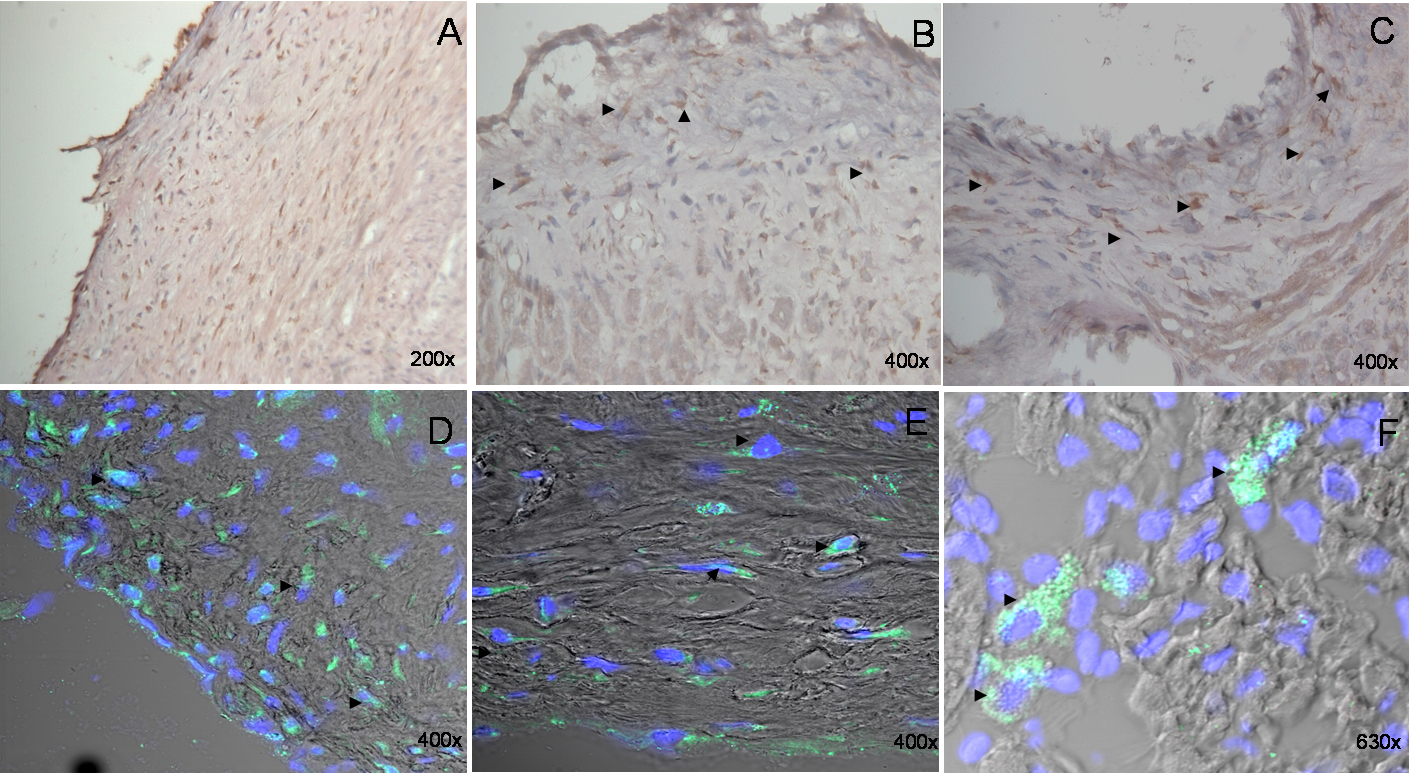

Supplement: Figure S2 — Immunodetection of GFP+ASCs four weeks after transplantation. Upper panel (A, B and C): immunohistochemistry. Bottom panel (D, E and F): immunofluorescence at infarction border zone. Arrowheads show the GFP+ASCs. Magnification 400×. (2.04 MB TIF) [file pone.0012077.s002.tif]
