# Supplementary material for: Rat Adipose Tissue-Derived Stem Cells Transplantation Attenuates Cardiac Dysfunction Post Infarction and Biopolymers Enhance Cell Retention
Source: PLoS One. 2010 Aug 10;5(8):e12077. doi: 10.1371/journal.pone.0012077 (PMC2919414; doi:10.1371/journal.pone.0012077)
Supplement: Methods S1 — (0.02 MB DOC) [file pone.0012077.s003.doc]

**SUPPLEMENTARY METHODS**

**Immunoassays for identification of GFP^+^ ASCs**

Frozen cardiac sections in Tissue-Tek OCT compound (Tissue-Tek®, Sakura Finetek, U.S.A. Inc. Torrance, CA) were first fixed in absolute acetone for 10 minutes and then blocked with 2% casein solution in PBS (Sigma-Aldrich). Tissue sections were then incubated with rabbit anti-GFP antibody (1:50, Santa Cruz) overnight at 4° C. Sections were incubated with a fluorescent secondary antibody (goat anti-rabbit Alexa fluor dye 488 1:600, Molecular Probes) for 90 minutes at 4° C and then with DAPI (1:100, Molecular Probes) according to manufacturer’s instructions. Negative control slides were incubated with secondary antibody alone. For immunohistochemistry, an HRP-conjugated goat anti-rabbit secondary antibody (Zymed Laboratories) was used. A total of 3 tissue sections from each group were used to identify the GFP^+^ cells.
